# Comparison of methods for the analysis of airway macrophage particulate load from induced sputum, a potential biomarker of air pollution exposure

**DOI:** 10.1186/s12890-015-0135-7

**Published:** 2015-11-05

**Authors:** Hannah Jary, Jamie Rylance, Latifa Patel, Stephen B. Gordon, Kevin Mortimer

**Affiliations:** Liverpool School of Tropical Medicine, Liverpool, UK; Aintree University Hospital, Liverpool, UK

**Keywords:** Air pollution, Particulate matter, Biomarker, Induced sputum, Airway macrophages

## Abstract

**Background:**

Air pollution is associated with a high burden or morbidity and mortality, but exposure cannot be quantified rapidly or cheaply. The particulate burden of macrophages from induced sputum may provide a biomarker. We compare the feasibility of two methods for digital quantification of airway macrophage particulate load.

**Methods:**

Induced sputum samples were processed and analysed using ImageJ and Image SXM software packages. We compare each package by resources and time required.

**Results:**

13 adequate samples were obtained from 21 patients. Median particulate load was 0.38 μm^2^ (ImageJ) and 4.0 % of the total cellular area of macrophages (Image SXM), with no correlation between results obtained using the two methods (correlation coefficient = −0.42, *p* = 0.256). Image SXM took longer than ImageJ (median 26 vs 54 mins per participant, *p* = 0.008) and was less accurate based on visual assessment of the output images. ImageJ’s method is subjective and requires well-trained staff.

**Conclusion:**

Induced sputum has limited application as a screening tool due to the resources required. Limitations of both methods compared here were found: the heterogeneity of induced sputum appearances makes automated image analysis challenging. Further work should refine methodologies and assess inter- and intra-observer reliability, if these methods are to be developed for investigating the relationship of particulate and inflammatory response in the macrophage.

## Background

Indoor and outdoor air pollution are the 4^th^ and 9^th^ leading risk factors, respectively, for disability-adjusted life years worldwide [1], and exposure is associated with increased risk of pneumonia in children, respiratory cancers, and development of Chronic Obstructive Pulmonary Disease [2–5]. Airborne particulate matter [6] with an aerodynamic diameter of <2.5 μm (PM_2.5_) is considered particularly harmful as the small size allows inhalation deep into the lungs [7].

Global initiatives, such as the Global Alliance for Clean Cookstoves (www.cleancookstoves.org), are tackling the major health burden caused by airborne PM. Major randomised trials of the health effects of clean burning cookstoves are in progress (e.g. www.capstudy.org and http://www.kintampo-hrc.org/projects/graphs.asp#.VMtKusaI0Rk). All share the challenge that quantifying an individual’s exposure to pollution is complex and expensive, and there is no gold standard method [8].

Development of a biomarker that acts as a surrogate marker of exposure could obviate the need for costly and intensive exposure monitoring. Ideally a biomarker should be: closely associated with exposure, adequately sensitive and specific, consistent across heterogenous populations, cost efficient, acceptable to the user population, and feasible for use in the field (including low-resource settings) [9].

The phagocytic action of airway macrophages (AM) may provide the basis for a biomarker of PM exposure. The particulate load within AM is: increased in individuals who report exposure to household air pollution compared to those who do not [10]; statistically different between individuals who use different types of domestic fuel [11]; and associated with exposure to outdoor PM in commuters who cycle in London [12]. Correlation between AM particulate load (AMPL) and worsening lung function supports a possible pathophysiological role [13]. A recent systematic review of studies calculating AMPL concluded that this biomarker is suitable for assessing personal exposure to PM, but that technical improvements are needed before this method is suitable for widespread use [14].

Once cell monolayers (Cytospins™) have been obtained from induced sputum (IS) or bronchoalveolar lavage (BAL) samples, several different digital image analysis software programmes can be used to calculate AMPL. ImageJ software (http://rsbweb.nih.gov/ij/, superseding a similar software, Scion Image) and Image SXM software [15] (http://www.ImageSXM.org.uk) have both been used for this purpose [12, 16, 17].

There is no previously reported objective comparison of their feasibility and it is unknown whether these two methods provide comparable results. Unlike ImageJ, Image SXM has only been used with samples obtained via BAL, a technique that is not suitable for widespread use in the field due to the expertise, risks and financial costs involved. This study therefore aimed to provide an objective assessment of the relative feasibilities – with regard to resources, expertise and time required - of ImageJ and Image SXM for use with IS samples, and their comparative accuracy.

## Methods

### Participant involvement

Respiratory patients were recruited via outpatient respiratory clinics at Aintree University Hospital, Liverpool, UK. All consenting adults over 18 years old with asthma or bronchiectasis, who did not meet safety exclusion criteria (see Table 1), were recruited.

**Table 1:** The exclusion criteria used for safety reasons prior to performing sputum induction

Safety checklist – exclusion criteria for sputum induction
• FEV_1_ < 60 %/< 1.0 L (post – Salbutamol 200 micrograms)
• SaO_2_ < 90 % on room air
• Unable to take salbutamol
• Extreme shortness of breath
• Acute Respiratory Distress Syndrome
• Known haemoptysis
• Known arrhythmias/angina
• Known thoracic, abdominal or cerebral aneurysms
• Recent pneumothorax
• Pulmonary emboli
• Fractured ribs/recent chest trauma
• Recent eye surgery
• Known pleural effusions
• Pulmonary oedema

Thrombocytopenia (Platelets < 25)

### Sputum induction

Participants underwent sputum induction on one occasion each in August-October 2013. Pre-procedure Salbutamol (200 micrograms) was given to prevent bronchoconstriction. Baseline spirometry was performed to European Respiratory Society and American Thoracic Society standards [18] using a MicroMedical MicroLab Mk8 Spirometer (Cardinal Health UK). Three × 5mls of hypertonic saline (3 %, 4 %, 5 % saline given in stepwise fashion, lasting up to 5 min per nebulisation) were nebulised via Omron NE-U17 Ultrasonic Nebuliser (Omron Healthcare Europe). Lung function was assessed at intervals to detect bronchoconstriction, according to pre-specified safety criteria.

### Sputum processing

Sputum samples were kept on ice and sputum plugs were manually extracted and treated with 0.1 % Sputolysin (Merck Chemical Ltd, UK) for fifteen minutes to remove mucus. Phosphate Buffered Solution (Sigma-Aldrich, UK) was added and cells were filtered and centrifuged at 2200 rpm for ten minutes at 4 °C (Heraeus Megafuge 1.0R, ThermoFisher Scientific, USA). The pellet was re-suspended at 0.5×10^6^ cells per ml and two × 100 μl of suspension was cytocentrifuged (Shandon Cytopsin 4, ThermoFisher Scientific) onto microscope slides at 450 rpm for 6 min to produce three cytospins per participant. Slides were fixed in methanol and stored until staining. One slide per participant was stained using Hemacolor Staining kit (Merck-Millipore, Germany) for ImageJ analysis. One slide was stained using Hemacolor Solution 2 (eosin) only (dipped for 9 s), so that only the cytoplasm was stained (a method previously developed for optimising Image SXM analysis [16]). One slide per participant was stained with Diff-Quik (Dade Behring, Deerfield, IL, USA) for differential cell counts : 400 cells were counted per participant, using a Leica DM IL light microscope at ×40 magnification. Cytospins with a leukocyte/squamous epithelial cell ratio of ≤5 were deemed inadequate and therefore excluded from the analysis [19].

### Digital image acquisition

**Fig. 1:**
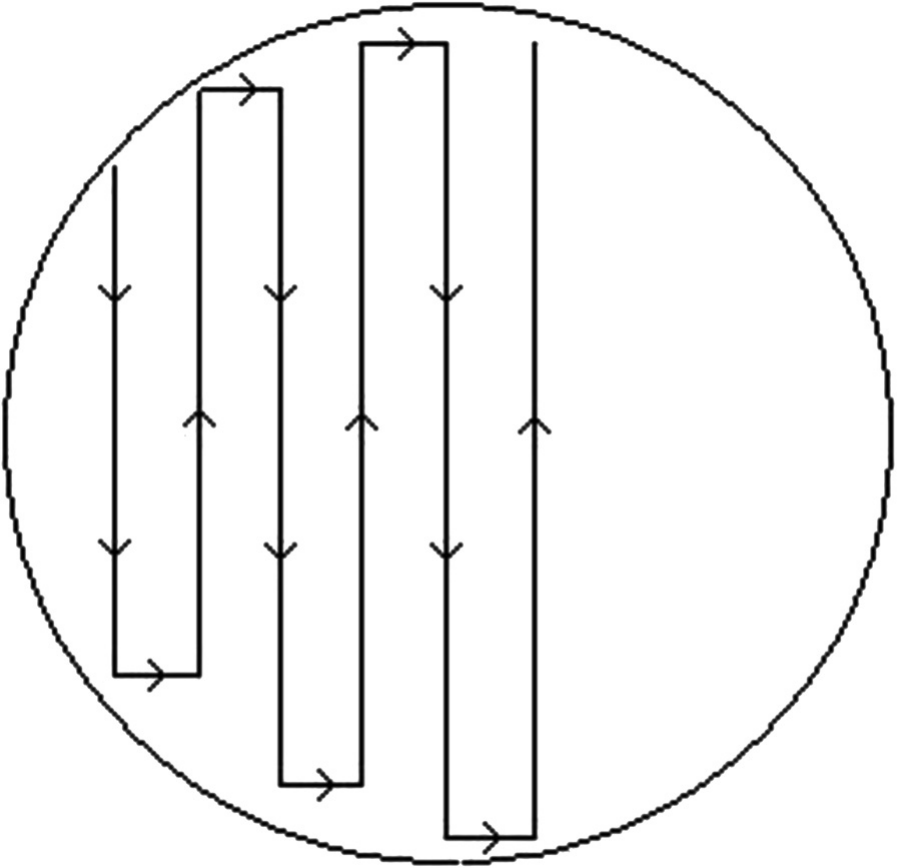
Systematic digital image acquisition. The pathway used to acquire digital images of cytospin ‘spots’ is shown

Cytospin slides for ImageJ analysis were photographed at ×60 magnification using Nikon Eclipse 80i digital microscope (Nikon Instruments Europe BV) with Nikon NIS-Elements BR software; 50 macrophages were captured per participant where possible (in cases where less than 50 macrophages were present on the cytospin a reduced number was used). Slides for Image SXM analysis were imaged at ×40 magnification using a Leica DM IL light microscope (Leica Microsystems UK Ltd) with a Nikon E990 digital camera (Nikon Inc, USA); where possible 50 microscope fields (with at least one macrophage per field) were captured per participant - all the macrophages captured in a field were analysed. In cases where less than 50 images from the whole cytospin contained a macrophage this reduced number of macrophages-containing images, and all macrophages within those images, were included in the analysis. Images for both methods were taken systematically using a predefined method to prevent duplication or biased image selection, as shown in Fig. 1.

### Image SXM analysis

**Fig. 2:**
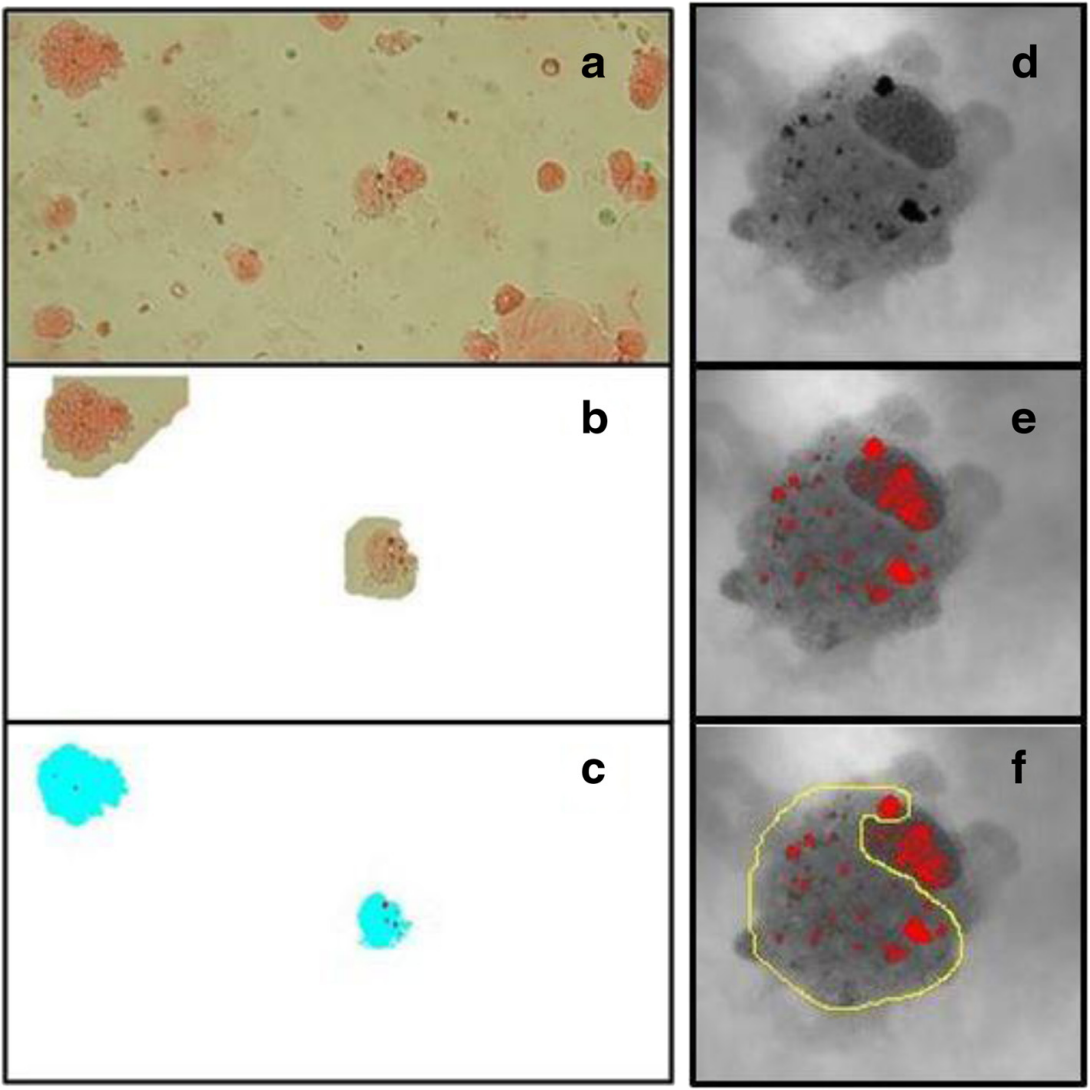
Image SXM and ImageJ methodology. Image SXM (**a**, **b** & **c**); digital images of the cytospins (**a**) were manually edited to remove all non-macrophage cells and debris (**b**). Image SXM then calculated the area of cytoplasm [27] and particulate matter (red), mapped out in the output image (**c**). ImageJ (**d**, **e** & **f**): for each macropghage, the threshold level was adjusted manually until the black areas of particulate matter seen in the original image (**a**) turned red (**b**). The particulate matter within the cytoplasm was then selected by freehand (**c**)

Images were edited using Adobe Photoshop Elements v6.0 to show only macrophages, to prevent incorrect calculations of cellular and PM areas (Fig. 2a and b). Image SXM (version 1.92, April 2011) variable settings were optimised for cytoplasm (upper and lower size limits and density threshold) and PM (density threshold) detection by adjusting settings for a range of images from different participants. Values which consistently maximised identification of PM without increasing false positive identification were used. These settings were then applied to the analysis of all images from all participants. 50 images per participant were analysed to generate output images (Fig. 2c) and the arithmetic mean percentage of total cellular area occupied by PM was calculated by Image SXM. The blink comparison function, which provides an overlay of images, was used to compare original and output images; subjective discordance between total cellular or PM area led to removal of that image from the analysis. Participants with fewer than ten images remaining were excluded from the analysis.

### ImageJ analysis

A stage micrometer (Agar Scientific, UK) was used to calibrate image size. Colour images were converted to 32-bit black and white images using ImageJ (version 1.46r). The “threshold” settings were adjusted to obtain the best fit of red over black areas [6] (Fig. 1d and e). The freehand select function was used to select PM (Fig. 2f) that was within the cell, and to exclude red areas other than PM, such as nucleus. ImageJ calculated the area of PM within the selection. Thresholds were adjusted to obtain the best fit for different particle aggregates in each macrophage. The median area from 50 macrophages was calculated. This methodology is a refinement of previously used techniques [12], adapted from earlier Scion Image methodology [10].

### Feasibility comparison of methods

The time taken for image capture and analysis of the final 11 samples was recorded, along with an inventory of the required equipment and expertise for each method.

### Statistical analysis

Data was analysed using SPSS v21. AMPL given by each method were compared using a Spearman Rank Order Correlation test. Participant characteristics were compared using Chi-square and Mann–Whitney U tests. Time taken to conduct the analyses was compared by Wilcoxon Signed Rank test. A p value of <0.05 was considered statistically significant.

### Ethical approval

The East Midlands – Derby 1 Research Ethics Committee approved this work (REC reference: 11/EM/0269). Written informed consent was obtained from all participants.

## Results

### Sputum induction

**Fig. 3:**
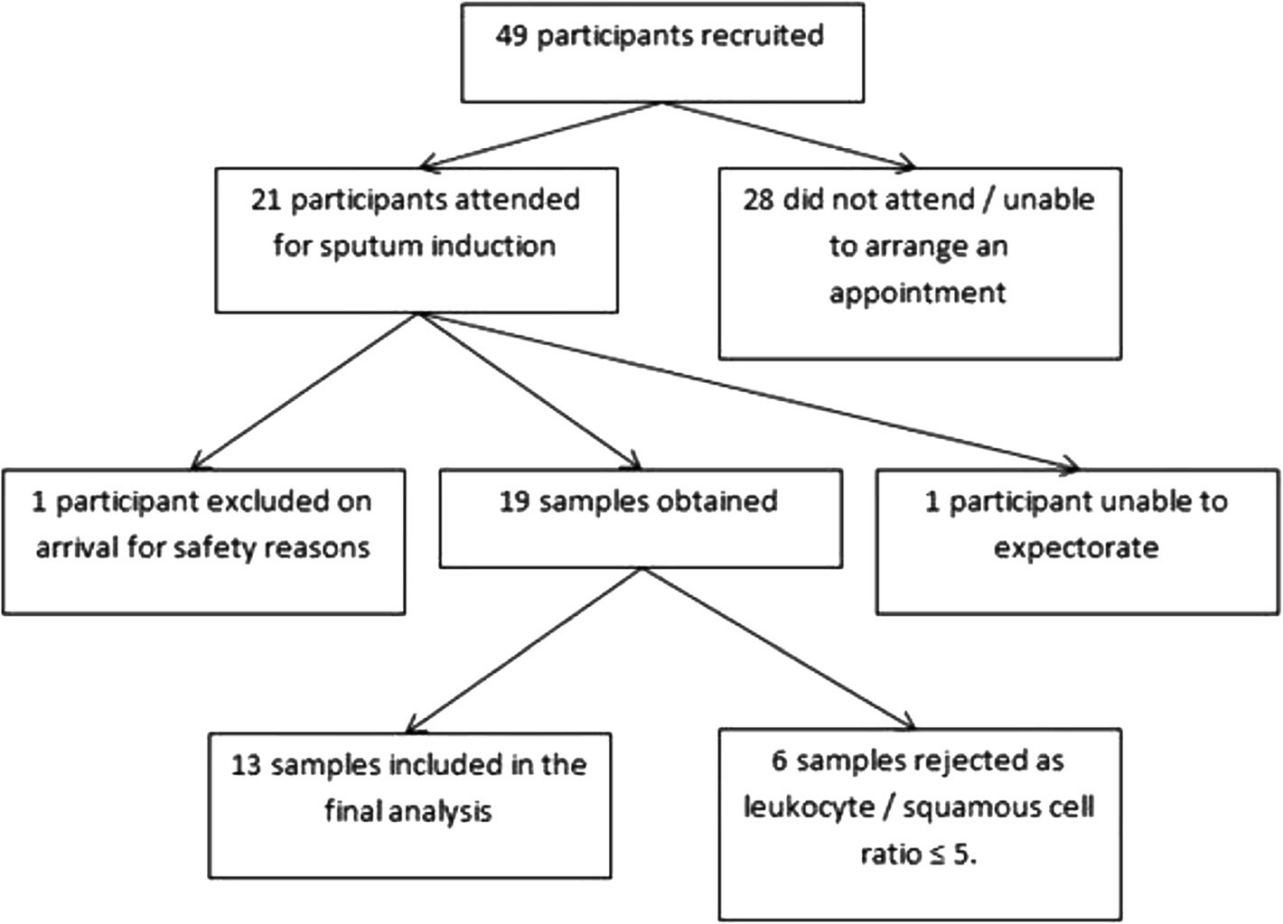
Participants and samples. The flow chart shows the number of consented and recruited patients, and how many samples were obtained and included in the final analysis

21 participants were recruited and attended for sputum induction and 1 participant was excluded due to baseline hypoxia (28 other recruited participants failed to attend). Of 20 participants undergoing sputum induction, samples were successfully obtained from 19 (Fig. 3). No adverse events occurred. Cytospins from six (32 %) participants were inadequate due to their leukocyte/squamous epithelial cell ratio. The characteristics of the 13 participants who provided an adequate sample are shown in Table 2. There was no significant difference in characteristics between those who provided an adequate sample and those who did not (data not shown). The differential cell counts are shown in Table 3.

**Table 2:** Characteristics of 13 participants

Participant characteristic		
Gender	Male, n (%)	9 (69)
	Female, n (%)	4 (31)
Age	Years, median (IQR)	57 (39–67)
Respiratory diagnosis	Asthma, n (%)	8 (62)
	Bronchiectasis, n (%)	2 (15)
	Both, n (%)	3 (23)
Smoking status	Never smoked (%)	8 (62)
	Ex-smoker (%)	5 (38)
Spirometry	FEV_1_, median (IQR), litres	1.80 (1.47-2.26)
	FEV_1_ % Predicted, median (IQR)	73.5 (60.1 - 77.6)
	FVC, median (IQR), litres	2.8 (2.47 – 3.82)
	FVC % predicted, median (IQR)	91.2 (87.6 – 109.0)

*IQR* Interquartile Range

**Table 3:** Differential cell counts

Cell type	Cell count % (Median (IQR) of 13 participants)
Neutrophil	72.5 (51.1-90.1)
Macrophage	10.0 (4.1-25.8)
Eosinophil	1.6 (1.0-8.5)
Lymphocyte	2.3 (1.2-3.5)
Metachromatic	0.0 (0.0-0.0)
Bronchial epithelial	2.8 (1.1-12.6)
Squamous epithelial	2.3 (0.8-6.5)

### Feasibility of methodology

Median time for analysis of each participant was significantly lower for Image J (26 mins, interquartile range (IQR): 21–30) than for Image SXM (54 mins, IQR: 43–68), *p* = 0.008. Including the time taken for image acquisition, the median time was not significantly different between ImageJ (51 mins,IQR: 46–65 mins) and Image SXM (66 mins, IQR: 59 – 84), *p* = 0.424. For the Image SXM method, 58 % of the ‘analysis time’ was spent editing the images prior to analysis. A comparison of the resources required for each method is shown in Table 4.

**Table 4:** Comparison of resource requirements for methods

Resource	Image SXM	ImageJ
Equipment required for sputum induction and sample processing	Identical specialist equipment and facilities required regardless of analysis method	
Image acquisition equipment	Microscope with x40 objective and digital image capturing capabilities	Microscope with x100^a^ objective and digital image capturing capabilities
Analysis software availability	In the public domain – available free of charge	In the public domain – available free of charge
Additional image editing software	Purchase required	Not required
Operating system for analysis software	Compatible with Mac operating systems	Compatible with Mac and Windows operating systems
File type availability	TIFF	JPEG, TIFF, GIF, BMP, DICOM, FITS and ‘raw’
Time required for sputum induction and processing	Approximately 90–120 min per participant	
Time required for image acquisition (median)	15 min	27 min
Time required for image analysis (including image editing if required) (median)	54 min	26 min

^a^Although a x100 objective is recommended for ImageJ methodology, a x60 objective was used in this study due to resource limitations

**Fig. 4:**
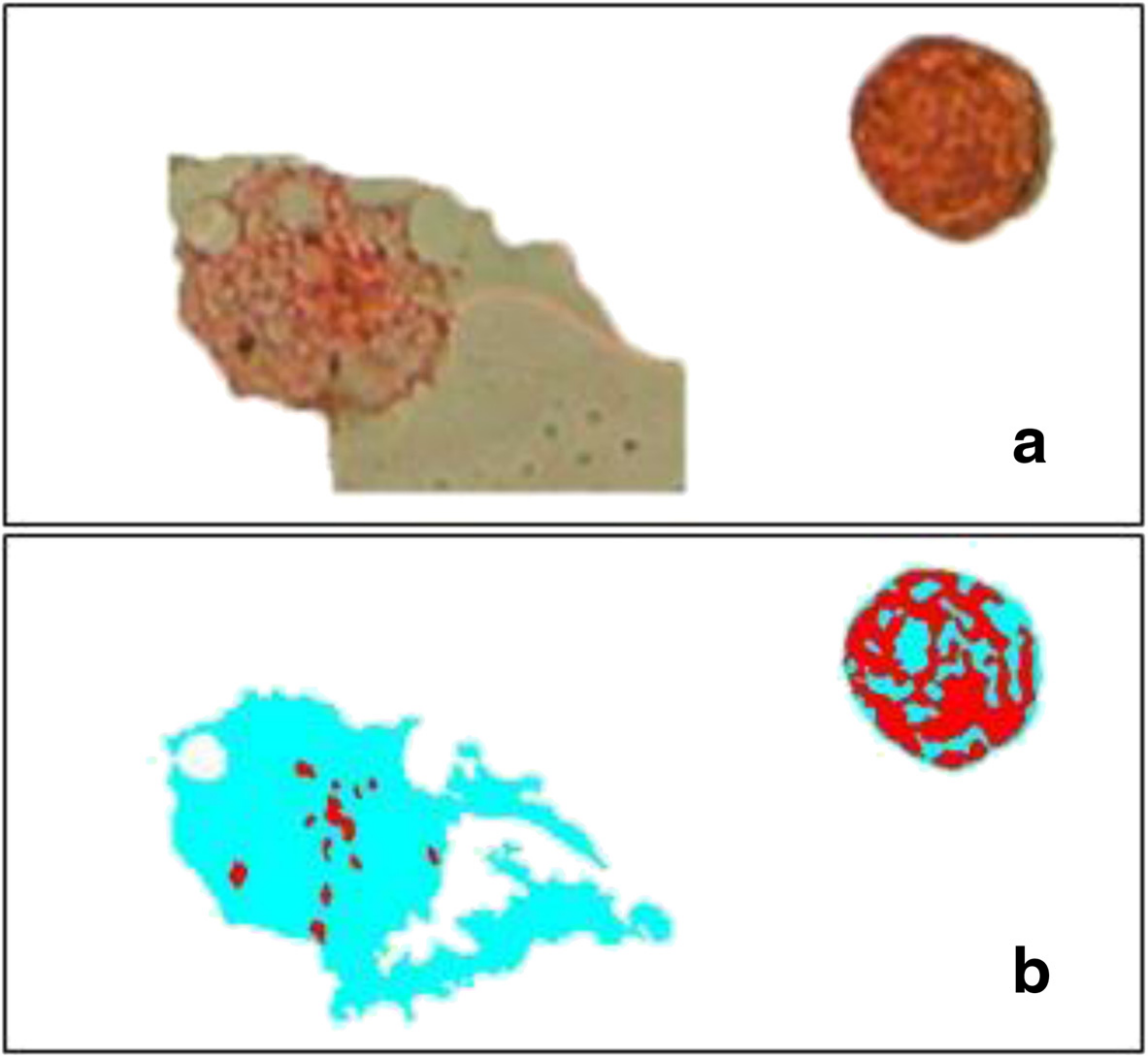
An example of inaccurte Image SXM analysis. Comparing the original image (**a**) to the output image (**b**), the total cellular area [27] of the airway macrophage on the left has been overestimated, and the partcilate matter (red) of the airway macrophage on the right has been overestimated

A mean of 49 macrophages per participant were included in the ImageJ analysis (total 632 macrophages). A mean of 43 images of per participant were captured for Image SXM analysis (total 558 images). During the Image SXM process, 72 % of images were removed following the initial analysis as they were deemed to be inaccurate (either over- or under-estimating AMPL) using the blink comparison function (Fig. 4), resulting in a further four participants being excluded from the study. The analysis was repeated with only the remaining 143 images (median 14 images (IQR 11.5-20) per participant). If only these nine participants are included, median time taken increased to 67 mins (IQR 47–72) for Image SXM analysis and 83 mins (IQR 64–87) including image acquisition time.

### Airway macrophage particulate load

**Fig. 5:**
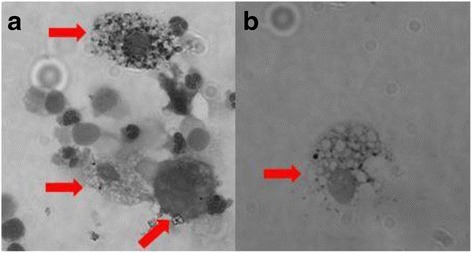
Airway macrophage heterogeneity. The morphology of the airway macrophages (shown with red arrows) was varied within the same sample (**a**) and between different participant samples (**a** & **b**). The particulate load also varied between macrophages in the same sample (**a**)

Considerable morphological heterogeneity was seen between AM, both within samples and between participants, with wide variations in AMPL (Fig. 5). The cytoplasms of the AM in this study were noted to be granular and heterogeneous (Fig. 5), unlike the homogenous appearance of cytoplasm seen in our previous experience of macrophages obtained by BAL [11].

ImageJ analysis of 13 cytospins revealed a median AMPL of 0.38 μm^2^ (IQR 0.17-0.72 μm^2^). Image SXM analysis of 9 cytospins calculated a median total cellular area occupied by PM of 4.0 % (IQR 2.3-6.0 %). There was no statistically significant correlation between results obtained using the two methods (correlation coefficient = −0.42, *p* = 0.256).

## Discussion

A biomarker which can be used in the field to assess an individual’s air pollution exposure will be a valuable tool for research into the health effects and benefits of interventions. In our pilot work for the Cooking and Pneumonia Study (www.capstudy.org) we identified the need for a biomarker representative of household air pollution exposure [8]. This study set out to explore the feasibility of using IS samples for assessment of AMPL as a potential biomarker.

Although the procedure was well-tolerated by all participants who underwent IS, there was a low appointment attendance rate despite multiple appointments being offered at their convenience. This may be due to participant’s availability, but may also reflect an unwillingness to undergo the procedure suggesting that IS may not be acceptable to the wider community. A third of participants were unable to produce adequate samples. These factors resulted in a small samples size, a major limitation of this study, but also reflects a potential limitation in the feasibility of using IS as a biomarker.

The time taken for the Image SXM method was substantially lengthened by the need to manually edit images prior to analysis to improve accuracy. This editing is not required when using this software with BAL samples, which tend to have few other cells or debris.

ImageJ was the quicker method for image acquisition and analysis (median 51 min). Image capturing software used in this study for the ImageJ method delayed this process by approximately 15 min, but was not used for the Image SXM method – a limitation of this study due to the lack of available equipment. However, when combined with the time taken for sputum induction and processing (usually >90 min), this process is unlikely to be feasible for widespread use in large studies given the total time required (>2 h per participant).

Both methods require considerable expenditure for clinical and laboratory equipment. Previously published studies using ImageJ method report using a microscope with a x100 objective, while the Image SXM method requires a x40 objective, both with digital image acquisition capabilities. In this study a x60 objective was used for the ImageJ method, as greater magnification was not available with digital image capturing capabilities. Although this may have theoretically reduced the accuracy of the ImageJ methodology in our study, we experienced no difficulties visualising particulate matter within the macrophages and still found ImageJ to be the more reliable of the two methods for detecting PM. As we do not comment on the accuracy of the ImageJ method in comparison to a gold standard assessment of exposure, this limitation of our study does not have a major impact on our findings. However, it does emphasise the need for specialised equipment, which has implications for feasibility.

Both softwares are available free of charge but ImageJ is more widely compatible. Image editing software must be also purchased if using Image SXM with IS. The facilities and equipment required for inducing and processing sputum are likely to preclude the use of this technique in rural or resource poor settings.

A further limitation of this study is that image capture of macrophages – which can be difficult to differentiate from other cell types (particularly on cytospins stained only with eosin for Image SXM analysis) - was only performed by one reader, with support from a senior cell biologist, without a priori criteria for inclusion. This may have resulted in incorrect identification of some cells. Independent image capture and slide analysis by two individuals with a high level of expertise may improve accuracy of macrophage identification, although this represents an additional challenge for implementing these methods in resource limited settings.

ImageJ method requires higher levels of operator training for image analysis than Image SXM, due to the subjective nature of the analysis process. Further work to assess intra- and inter-observer reliability using the ImageJ method is required before this is widely used – this was not evaluated as part of this study in which only one unblinded reader performed the analysis.

Although previously successfully used with BAL samples, Image SXM appears to not perform as well with IS macrophages. This is possibly due to the heterogenous and granular nature of these macrophages making it difficult for the software to distinguish between cytoplasm and PM, as has been observed in previous studies [14]. We postulate that the difference in appearance compared to BAL macrophages is either due to these being a different population of macrophages, taken from a more proximal part of the airways, or due to cell stress or apoptosis resulting from the IS process, although we did not measure cell viability in this study. Steps were taken to ensure threshold settings were optimised for this batch of images, but due to the heterogeneity seen these settings were not always optimal for each individual image. Image SXM does include an option to adjust the threshold settings manually for different images. This might improve accuracy but would make the process more time-consuming, and would not account for heterogeneity of macrophages within the same image (Fig. 5). Optimising the threshold settings for each image might reduce the number of images discarded from Image SXM following visual checking for accuracy (Fig. 4). This might increase the sample size and therefore the precision of estimates.

The lack of correlation observed in AMPL results between the two methods is unsurprising given some of the difficulties outlined above. To determine the accuracy of either method, comparison with an external comparator is required, such as an individual’s PM exposure data. This, and assessment of intra- and inter-observer reliability, were beyond the scope of this study. An association between AMPL calculated and the number of peak exposures to PM has been demonstrated in London cyclists [20], but further exploration of this relationship in other settings is required. The results obtained by the ImageJ method in this study are comparable to that of healthy British children (0.41 μm_2_ PM per macrophage) [13]. Other studies using ImageJ methodology have suggested that AMPL does correlate with exposure [10, 13].

Given the fundamental role of alveolar macrophages in the defence against inhaled pollutants, further exploration of the relationship between AMPL and pathophysiology is an intuitive way to improve understanding of the health impacts of air pollution. Optimising digital analysis software or using alternative methods for quantifying AMPL, such as spectrophotometry, may assist with this, but is unlikely to provide a useful field biomarker of exposure.

## Conclusion

Direct measurement of air pollution exposure is costly, logistically complicated and intrusive to the individual. Studies investigating the health impacts of air pollution exposure and the benefits of interventions are limited by the challenges associated with accurately quantifying exposure [9]. A biomarker of air pollution exposure will be a useful tool to facilitate research addressing the high burden of disease associated with air pollution. This small study has not established whether AMPL is an accurate biomarker of pollution exposure, but has compared the feasibility of two previously used methods. The heterogeneity of IS samples complicates digital image analysis methods, and the resource requirements for assessing AMPL from IS are considerable, making it unlikely that this biomarker of exposure will be appropriate for widespread use as a tool for large-scale intervention studies. Priority should be given to developing a point-of-care biomarker of exposure, without the need for specialist training and equipment, to facilitate the large public health intervention trials that are urgently needed. Potential biomarkers requiring further exploration include direct measures of combustion products, such as exhaled carbon monoxide, exhaled carboxyhaemoglobin, exhaled volatile organic compounds or levoglucosan and methoxyphenols in urine [8, 9, 21–23]. Indirect measures of exposure in sputum, blood and urine, including markers of oxidative stress and endothelial or epithelial damage (such as 8-isoprostane, malondialdehyde, nitric oxide, or surfactant-associated protein D), may also be promising biomarkers [9, 21, 24–26].

## Abbreviations

AM: airway macrophage
AMPL: airway macrophage particulate load
BAL: bronchoalveolar lavage
IQR: interquartile range
IS: induced sputum
PM: particulate matter
PM_2.5_: particulate matter with a diameter less than 2.5 μm
REC: research ethics committee

## References

[1] Lim SS, Vos T, Flaxman AD, Danaei G, Shibuya K, Adair-Rohani H, Amann M, Anderson HR, Andrews KG, Aryee M (2013). A comparative risk assessment of burden of disease and injury attributable to 67 risk factors and risk factor clusters in 21 regions, 1990–2010: a systematic analysis for the Global Burden of Disease Study 2010. Lancet.

[2] da Silva LF, Saldiva SR, Saldiva PH, Dolhnikoff M (2012). Impaired lung function in individuals chronically exposed to biomass combustion. Environ Res.

[3] Dherani M, Pope D, Mascarenhas M, Smith KR, Weber M, Bruce N (2008). Indoor air pollution from unprocessed solid fuel use and pneumonia risk in children aged under five years: a systematic review and meta-analysis. Bull World Health Organ.

[4] Gordon SB, Bruce NG, Grigg J, Hibberd PL, Kurmi OP, Lam KB, Mortimer K, Asante KP, Balakrishnan K, Balmes J (2014). Respiratory risks from household air pollution in low and middle income countries. Lancet Res Med.

[5] Mehta S, Shin H, Burnett R, North T, Cohen AJ (2013). Ambient particulate air pollution and acute lower respiratory infections: a systematic review and implications for estimating the global burden of disease. Air QualityAtmosphere Health.

[6] Shen M, Chapman RS, Vermeulen R, Tian L, Zheng T, Chen BE, Engels EA, He X, Blair A, Lan Q (2009). Coal use, stove improvement, and adult pneumonia mortality in Xuanwei, China: a retrospective cohort study. Environ Health Perspect.

[7] Yu CP, Xu GB (1987). Predictive models for deposition of inhaled diesel exhaust particles in humans and laboratory species. Res Rep Health Eff Inst.

[8] Jary HR, Kachidiku J, Banda H, Kapanga M, Doyle JV, Banda E, Fox C, Gordon SB, Mortimer K (2014). Feasibility of conducting a randomised controlled trial of a cookstove intervention in rural Malawi. Int J Tuberc Lung Dis.

[9] Rylance J, Gordon SB, Naeher LP, Patel A, Balmes JR, Adetona O, Rogalsky DK, Martin WJ (2013). Household air pollution: a call for studies into biomarkers of exposure and predictors of respiratory disease. Am J Physiol Lung Cell Mol Physiol.

[10] Kulkarni NS, Prudon B, Panditi SL, Abebe Y, Grigg J (2005). Carbon loading of alveolar macrophages in adults and children exposed to biomass smoke particles. Sci Total Environ.

[11] Fullerton DG, Jere K, Jambo K, Kulkarni NS, Zijlstra EE, Grigg J, French N, Molyneux ME, Gordon SB (2009). Domestic smoke exposure is associated with alveolar macrophage particulate load. Trop Med Int Health.

[12] Nwokoro C, Ewin C, Harrison C, Ibrahim M, Dundas I, Dickson I, Mushtaq N, Grigg J (2012). Cycling to work in London and inhaled dose of black carbon. Eur Respir J.

[13] Kulkarni N, Pierse N, Rushton L, Grigg J (2006). Carbon in airway macrophages and lung function in children. N Engl J Med.

[14] Bai Y, Brugha RE, Jacobs L, Grigg J, Nawrot TS, Nemery B (2014). Carbon loading in airway macrophages as a biomarker for individual exposure to particulate matter air pollution - A critical review. Environ Int.

[15] Barrett SD. Image SXM. In*.*, v1.92 edn; 2011 http://www.ImageSXM.org.uk. Accessed 28/06/2013.

[16] Fullerton DG (2011). Indoor Air Pollution from biomass fuel smoke and its effect of respiratory health, in a population at risk of HIV related pneumonia.

[17] Rylance J (2012). The role of redox balance in pulmonary innate immunity.

[18] Miller MR, Hankinson J, Brusasco V, Burgos F, Casaburi R, Coates A, Crapo R, Enright P, van der Grinten CP, Gustafsson P (2005). Standardisation of spirometry. Eur Respir J.

[19] Sadeghi E, Matlow A, MacLusky I, Karmali MA (1994). Utility of gram stain in evaluation of sputa from patients with cystic fibrosis. J Clin Microbiol.

[20] Nwokoro C, Brugha R, Grigg J (2013). Alveolar macrophages carbon load: a marker of exposure?. Eur Respir J.

[21] Miranda AI, Martins V, Cascao P, Amorim JH, Valente J, Borrego C, Ferreira AJ, Cordeiro CR, Viegas DX, Ottmar R (2012). Wildland smoke exposure values and exhaled breath indicators in firefighters. J Toxic Environ Health A.

[22] Torres-Dosal A, Perez-Maldonado IN, Jasso-Pineda Y, Martinez Salinas RI, Alegria-Torres JA, Diaz-Barriga F (2008). Indoor air pollution in a Mexican indigenous community: evaluation of risk reduction program using biomarkers of exposure and effect. Sci Total Environ.

[23] Bergauff MA, Ward TJ, Noonan CW, Migliaccio CT, Simpson CD, Evanoski AR, Palmer CP (2010). Urinary levoglucosan as a biomarker of wood smoke: results of human exposure studies. J Expo Sci Environ Epidemiol.

[24] Barregard L, Sallsten G, Gustafson P, Andersson L, Johansson L, Basu S, Stigendal L (2006). Experimental exposure to wood-smoke particles in healthy humans: effects on markers of inflammation, coagulation, and lipid peroxidation. Inhal Toxicol.

[25] Gong J, Zhu T, Kipen H, Wang G, Hu M, Ohman-Strickland P, Lu SE, Zhang L, Wang Y, Zhu P (2013). Malondialdehyde in exhaled breath condensate and urine as a biomarker of air pollution induced oxidative stress. J Expo Sci Environ Epidemiol.

[26] Van Miert E, Sardella A, Nickmilder M, Bernard A (2012). Respiratory effects associated with wood fuel use: a cross-sectional biomarker study among adolescents. Pediatr Pulmonol.

[27] Vasamreddy CR, Jayam V, Bluemke DA, Calkins H (2004). Pulmonary vein occlusion: an unanticipated complication of catheter ablation of atrial fibrillation using the anatomic circumferential approach. Heart Rhythm.

